# Hyperglycaemia Aggravates Oxidised Low-Density Lipoprotein-Induced Schwann Cell Death via Hyperactivation of Toll-like Receptor 4

**DOI:** 10.3390/neurolint16020027

**Published:** 2024-03-19

**Authors:** Wataru Nihei, Ayako Kato, Tatsuhito Himeno, Masaki Kondo, Jiro Nakamura, Hideki Kamiya, Kazunori Sango, Koichi Kato

**Affiliations:** 1 Laboratory of Medicine, Aichi Gakuin University School of Pharmacy, Nagoya 464-8650, Japan; nihei@dpc.agu.ac.jp (W.N.); k-ayako@dpc.agu.ac.jp (A.K.); 2 Division of Diabetes, Department of Internal Medicine, Aichi Medical University School of Medicine, Nagakute 480-1195, Japankondou.masaki.330@mail.aichi-med-u.ac.jp (M.K.); hkamiya@aichi-med-u.ac.jp (H.K.); 3 Department of Innovative Diabetes Therapy, Aichi Medical University School of Medicine, Nagakute 480-1195, Japan; nakamura.jirou.574@mail.aichi-med-u.ac.jp; 4 Diabetic Neuropathy Project, Department of Diseases and Infection, Tokyo Metropolitan Institute of Medical Science, Tokyo 156-8506, Japan; sango-kz@igakuken.or.jp

**Keywords:** diabetic neuropathy, TLR4, OxLDL, apoptosis

## Abstract

Increased low-density lipoprotein levels are risk factors for diabetic neuropathy. Diabetes mellitus is associated with elevated metabolic stress, leading to oxidised low-density lipoprotein formation. Therefore, it is important to investigate the mechanisms underlying the pathogenesis of diabetic neuropathy in diabetes complicated by dyslipidaemia with increased levels of oxidised low-density lipoprotein. Here, we examined the effects of hyperglycaemia and oxidised low-density lipoprotein treatment on Schwann cell death and its underlying mechanisms. Immortalised mouse Schwann cells were treated with oxidised low-density lipoprotein under normo- or hyperglycaemic conditions. We observed that oxidised low-density lipoprotein-induced cell death increased under hyperglycaemic conditions compared with normoglycaemic conditions. Moreover, hyperglycaemia and oxidised low-density lipoprotein treatment synergistically upregulated the gene and protein expression of toll-like receptor 4. Pre-treatment with TAK-242, a selective toll-like receptor 4 signalling inhibitor, attenuated hyperglycaemia- and oxidised low-density lipoprotein-induced cell death and apoptotic caspase-3 pathway. Our findings suggest that the hyperactivation of toll-like receptor 4 signalling by hyperglycaemia and elevated oxidised low-density lipoprotein levels synergistically exacerbated diabetic neuropathy; thus, it can be a potential therapeutic target for diabetic neuropathy.

## 1. Introduction

Diabetic neuropathy is the earliest and most common complication of diabetes, and a globally serious diabetes complication, since the global number of patients with diabetes was estimated to be 537 million in 2021, and up to half of the patients have peripheral neuropathy [1,2,3,4]. Although various mechanisms are involved in the onset and development of diabetic neuropathy, including abnormalities in polyol metabolism, protein kinase C pathway, and accumulation of advanced glycation end-products, additional yet unidentified mechanisms also play a role; thus, effective therapy for the management of diabetic neuropathy remains to be established [5,6,7,8]. Toll-like receptor 4 (TLR4), a receptor that recognises the cell surface component of gram-negative bacteria, including *Escherichia coli*, and plays a crucial role in innate immunity, is expressed in broad cell types, including smooth muscle, endothelial, neuronal, and glial cells [9,10,11,12,13]. TLR4 also recognises endogenous ligands such as oxidised low-density lipoprotein (oxLDL), which is LDL-modified via oxidation, leading to injury and death in various cell types [13,14,15]. Both TLR4 and oxLDL levels are elevated in diabetes, and an increase in circulating LDL, which is a precursor of oxLDL, is a risk factor for diabetic neuropathy [16,17,18,19]. However, the roles of TLR4 and oxLDL in the pathogenesis of diabetic neuropathy are not fully elucidated. Increased TLR4 expression in various tissues and LDL oxidation in the circulation proceed simultaneously under diabetic conditions; nevertheless, previous studies have investigated the effects of increased TLR4 or oxLDL alone but the combined effects of hyperglycaemia have not been examined [13,20]. Therefore, this study focused on the synergistic effects of hyperglycaemic conditions that mimic the diabetic state and oxLDL levels upon cell death in immortalised mouse Schwann (IMS32) cells.

## 2. Materials and Methods

### 2.1. Materials and Antibodies

3-(4,5-dimethyl-2-thiazolyl)-2,5-diphenyl-2H-tetrazolium bromide (MTT) was purchased from Dojindo (Kumamoto, Japan). Rabbit anti-TLR4 antibody was acquired from Cell Signalling Technology (cat # 14358S; Danvers, MA, USA), and mouse anti-β-actin antibody was purchased from Santa Cruz Biotechnology (cat # sc-69879; Dallas, TX, USA). Alexa 488-conjugated goat anti-mouse IgG was procured from Cell Signalling Technology (cat # 4408S). Horseradish peroxidase (HRP)-conjugated goat anti-rabbit antibody and HRP-conjugated horse anti-mouse antibody were purchased from Cell Signalling Technology (cat # 7074S, 7076S).

### 2.2. Cell Culture

IMS32 cells were provided by Dr. Kazuhiko Watabe (Kyorin University, Tokyo, Japan). The cells were cultured in low-glucose Dulbecco’s modified Eagle’s medium (Wako Pure Chemical Industries, Osaka, Japan) and supplemented with 5% foetal bovine serum (Gibco, Paisley, UK), 100 units/mL of penicillin, and 100 μg/mL of streptomycin, at 37 °C in a 5% CO_2_ incubator. For experiments, cells were cultured in either normoglycaemic (5.5 mM glucose) or hyperglycaemic (25 mM glucose) conditions.

### 2.3. Preparation of oxLDL

Human plasma LDL was purchased from Lee Biosolutions (Maryland Heights, MO, USA). LDL was oxidised with 20 μM CuSO_4_ in phosphate-buffered saline (PBS) at 37 °C for 24 h, as described previously [21]. The oxidation process was terminated by adding 1 mM of EDTA.

### 2.4. MTT Assay

IMS32 cells were cultured in 96-well plates. For TAK-242 treatment, cells were pre-treated with 100 nM TAK-242 for 2 h and incubated with oxLDL (0, 150, and 300 g/mL) for 24 h, followed by an MTT assay [22]. For the MTT assay, cells were treated with 0.5 mg/mL of MTT in the medium for 3 h at 37 °C in a 5% CO_2_ incubator. The resulting formazan was solubilised in dimethyl sulfoxide. At 535 nm, the absorbance was measured using a microplate reader (Tecan, Mannedorf, Switzerland). The cell viability was normalised to that of the control cells.

### 2.5. RNA Isolation, Quantitative Real-Time Polymerase Chain Reaction (PCR), and Electrophoresis

The IMS32 cells were seeded in 6-well plates and cultured overnight at 37 °C. The cells were incubated with or without oxLDL (150 μg/mL) in either normo- or hyperglycaemic conditions for 24 h, followed by RNA extraction. Total RNA was extracted using NucleoSpin^®^ RNA Plus (Takara Bio, Shiga, Japan). The first-strand cDNA was synthesised using the PrimeScript RT Reagent Kit (Takara Bio). PCR and quantitative real-time PCR were performed using Thunderbird Next SYBR qPCR Mix (TOYOBO, Osaka, Japan) with primers on a Takara Thermal Cycler Dice Real Time System III (Takara Bio) under the following conditions: initial degeneration at 95 °C for 30 s, amplification by 40 cycles of 95 °C for 15 s, and 60 °C for 60 s. mRNA expression levels were determined following normalisation to beta-actin using the 2^−ΔΔCt^ method [23]. The PCR products were subjected to electrophoresis and visualised using GelRed Nucleic Acid Gel Stain (Wako Pure Chemical Industries). The primers used are listed in Table 1.

### 2.6. Immunocytochemistry

For immunocytochemistry, the cells were seeded onto coverslips and cultured for 24 h (followed by fixation with 4% paraformaldehyde for 10 min at 25 °C), blocked with 1% bovine serum albumin containing 0.1% tween-20, incubated with anti-TLR4 primary antibody (1:200) for 1 h at 25 °C, washed with PBS, incubated with Alexa 488-conjugated secondary antibody (1:500) for 1 h at 25 °C, and mounted with Fluoroshield and DAPI (ImmunoBioScience Corp., Mukilteo, WA, USA). Cells were visualised using a confocal laser scanning microscope (LSM800, Carl Zeiss, Jena, Germany).

### 2.7. Western Blot

The IMS32 cells were seeded in 6-well plates and cultured overnight at 37 °C, followed by incubation with or without oxLDL (150 μg/mL) in either normo- or hyperglycaemic conditions for 24 h, and subjected to cell lysis for protein extraction. The cells were washed with PBS and lysed in radioimmunoprecipitation assay buffer (Wako Pure Chemical Industries) containing a protease inhibitor (Sigma-Aldrich; Merck KGaA, Darmstadt, Germany). The lysates were sonicated and centrifuged at 12,000 × *g* for 5 min at 4 °C, separated by gel electrophoresis, and transferred to polyvinylidene difluoride membranes (Sigma-Aldrich), which were subsequently incubated overnight at 4 °C with 1000-fold diluted anti-TLR4 and β-actin antibody, probed with 5000-fold diluted HRP-conjugated secondary antibody, and then detected with Clarity Max Western ECL Substrate or Clarity Western ECL Substrate (Bio-Rad, Hercules, CA, USA).

### 2.8. Caspase-3 Activity Detection

The IMS32 cells were seeded in 6-well plates and cultured overnight at 37 °C. For TAK-242 treatment, cells were pre-treated with 100 nM TAK-242 for 2 h and incubated with or without oxLDL (150 μg/mL) for 3 h. Caspase-3 activity was analysed using a Caspase-3 Assay Kit (ab39401, Abcam, Cambridge, UK) and normalised with the protein content.

### 2.9. Statistical Analysis

Statistical analyses were performed using SPSS software Ver. 27.0 (IBM, Chicago, IL, USA) or Statcel4 software Ver. 4 (OMS Publishing, Tokyo, Japan). The normality of distribution and homogeneity of variance of the data were confirmed with the Shapiro–Wilk test and Levene’s test, respectively. Data are expressed as mean ± standard error, and means were compared through a two-way analysis of variance, followed by Tukey’s post hoc test. A *p*-value < 0.05 was considered statistically significant.

## 3. Results

### 3.1. Hyperglycaemia and oxLDL Treatment Trigger Synergistic Cell Death in IMS32 Cells

**Figure 1:**
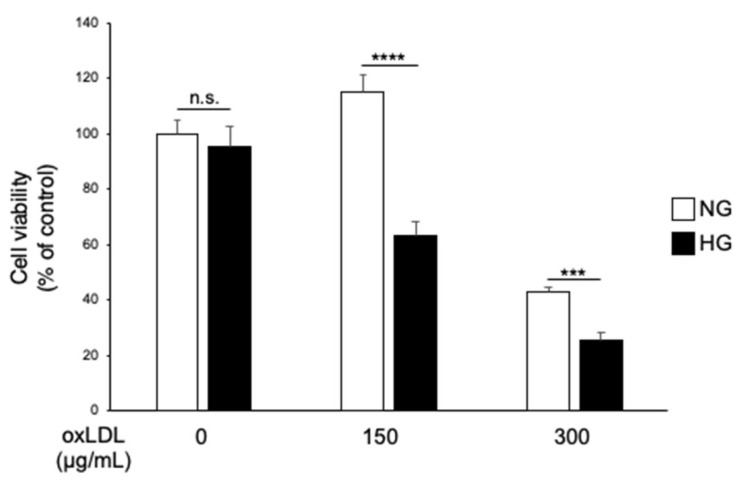
Hyperglycaemia potentiates oxLDL-induced cell death. IMS32 cells were cultured in either normoglycaemic (5.5 mM glucose) or hyperglycaemic (25 mM glucose) conditions in the presence of oxLDL (150 and 300 g/mL) for 24 h. Cell viability was determined by the MTT assay (*n* = 5 per group). Data are representative of at least three independent experiments, mean ± SE. *** *p* < 0.001, **** *p* < 0.0001, comparisons between indicated groups. IMS32, immortalised mouse Schwann; MTT, 3-(4,5-dimethyl-2-thiazolyl)-2,5-diphenyl-2H-tetrazolium bromide; n.s., not significant; oxLDL, oxidised low-density lipoprotein; SE, standard error.

The IMS32 cells were treated with oxLDL (150 or 300 g/mL) to evaluate the effect of hyperglycaemia on oxLDL-induced Schwann cell death under normo- (NG) or hyperglycaemic (HG) conditions, followed by an MTT cell viability assay. The cytotoxic effect of oxLDL treatment was observed to be higher under HG conditions than under NG conditions (Figure 1, 150 μg/mL NG versus 150 μg/mL HG, 114.9 ± 6.2 versus 63.2 ± 5.2; 300 μg/mL NG versus 300 μg/mL HG; 43.0 ± 1.5 versus 25.5 ± 2.9).

**Figure 2:**
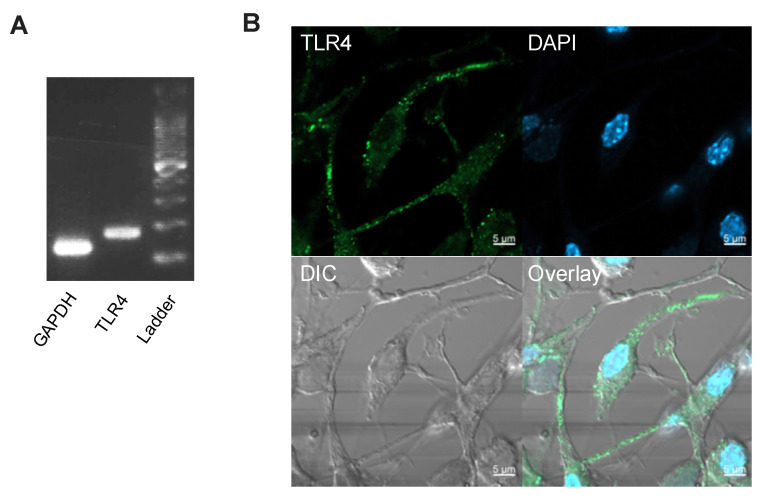
Expression and localisation of TLR4 in IMS32 cells. (**A**) TLR4 mRNA expression in IMS32 cells. (**B**) IMS32 cells were grown on coverslips for 24 h. The cells were fixed with 4% paraformaldehyde, permeabilised, and immunostained with anti-TLR4 antibody (green). The nuclei were stained with DAPI (blue). DIC, differential interference contrast scale bar, 5 μm.

These data indicated that hyperglycaemia potentiated oxLDL-dependent cell death in IMS32 cells. Next, we investigated possible receptors that mediate oxLDL-induced cytotoxicity. Using reverse transcription PCR and immunocytochemistry, we confirmed the expression of TLR4, the main receptor for oxLDL, in IMS32 cells (Figure 2).

### 3.2. Hyperglycaemia and oxLDL Treatment Upregulate TLR4 Gene and Protein Expression

**Figure 3:**
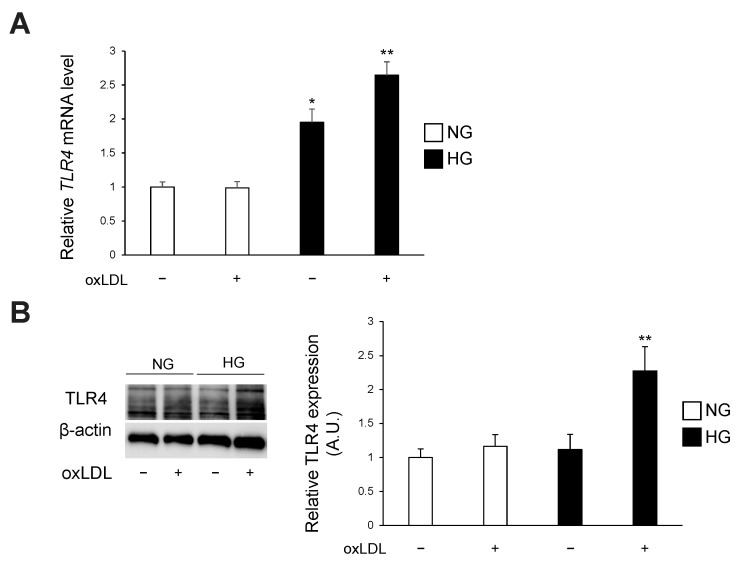
Hyperglycaemia and oxLDL synergistically upregulate TLR4 expression in IMS32 cells. IMS32 cells were cultured in either normo- or hyperglycaemic conditions in the presence or absence of oxLDL (150 μg/mL) for 24 h. (**A**) Relative mRNA expressions of TLR4 were tested by RT-qPCR (*n* = 6 per group). (**B**) Immunoblot analysis of TLR4 expression (*n* = 12 per group). Data are presented as the means of two (**A**) or three independent experiments. * *p* < 0.05, ** *p* < 0.01. IMS32, immortalised mouse Schwann; oxLDL, oxidised low-density lipoprotein; RT-qPCR, quantitative reverse transcription polymerase chain reaction; TLR4, toll-like receptor 4.

Subsequently, we tested the effect of hyperglycaemia on TLR4 expression in oxLDL-treated IMS32 cells. Hyperglycaemia alone or in combination with oxLDL treatment upregulated TLR4 gene expression, and the combined effect was synergistic (Figure 3A, NG oxLDL(−), NG oxLDL(+), HG oxLDL(−), HG oxLDL(+); 1.00 ± 0.07, 0.98 ± 0.09, 1.94 ± 0.19, 2.64 ± 0.19). Western blotting analysis demonstrated that the combination of hyperglycaemia and oxLDL treatment synergistically increased the protein expression of TLR4 (Figure 3B, NG oxLDL(−), NG oxLDL(+), HG oxLDL(−), HG oxLDL(+); 1.00 ± 0.12, 1.16 ± 0.17, 1.11 ± 0.22, 2.27 ± 0.35).

### 3.3. TLR4 Inhibition Attenuates Cell Death Caused by Hyperglycaemia and oxLDL Treatment

**Figure 4:**
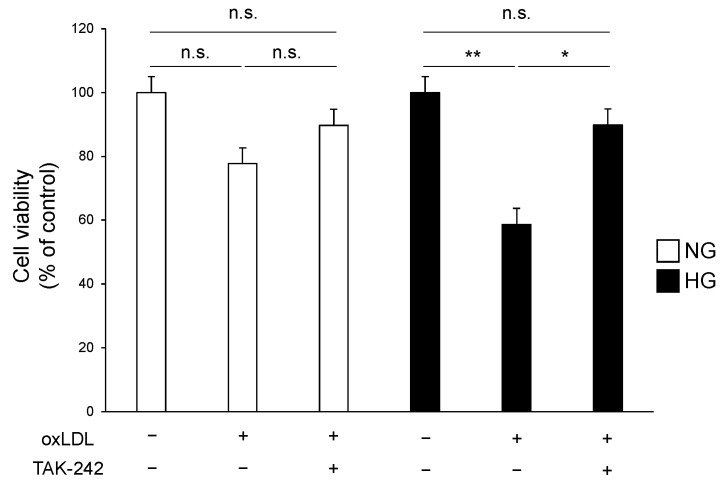
TLR4 inhibition attenuated the hyperglycaemia and oxLDL-mediated cell death. IMS32 cells were pre-treated with or without TAK-242 (100 nM) for 2 h and cultured in normo- or hyperglycaemic conditions in the presence or absence of oxLDL (150 μg/mL) for 24 h. Cell viability was quantified by the MTT assay (*n* = 5 per group). Data are representative of at least three independent experiments. * *p* < 0.05, ** *p* < 0.01, comparisons between indicated groups. IMS32, immortalised mouse Schwann; MTT, 3-(4,5-dimethyl-2-thiazolyl)-2,5-diphenyl-2H-tetrazolium bromide; n.s., not significant; oxLDL, oxidised low-density lipoprotein; TLR4, toll-like receptor 4.

We tested our hypothesis that the increase in cell death induced by the combination of hyperglycaemia and oxLDL treatment was due to the hyperactivation of the TLR4 pathway. The cells were treated with oxLDL under NG or HG conditions with or without pre-treatment with TAK-242, a selective TLR4 inhibitor, followed by an MTT cell viability assay. Under NG conditions, oxLDL treatment did not significantly induce cell death, and TAK-242 pre-treatment had no notable effect (Figure 4, NG oxLDL(−) TAK-242(−), NG oxLDL(+) TAK-242(−), NG oxLDL(+) TAK-242(+); 100 ± 4.9, 77.7 ± 5.6, 89.7 ± 8.0). In contrast, TAK-242 pre-treatment significantly attenuated oxLDL-dependent cell death under HG conditions (Figure 4, HG oxLDL(−) TAK-242(−), HG oxLDL(+) TAK-242(−), HG oxLDL(+) TAK-242(+); 96.6 ± 9.7, 56.7 ± 6.9, 86.8 ± 4.7).

### 3.4. TLR4 Inhibition Suppressed Hyperglycaemia and oxLDL-Induced Activation of Caspase-3 Pathway

**Figure 5:**
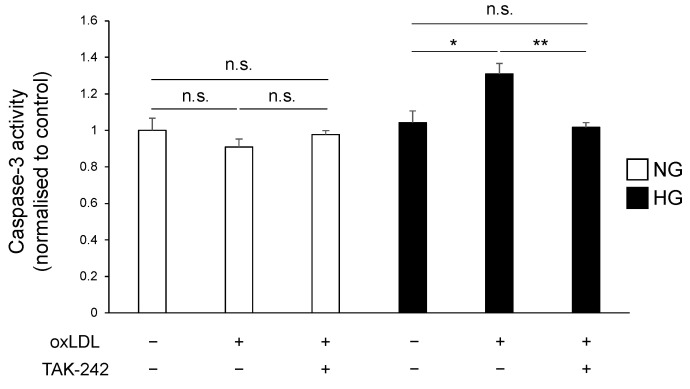
TLR4 inhibition suppressed caspase-3-dependent apoptosis. IMS32 cells were pre-treated with or without TAK-242 (100 nM) for 2 h and cultured in either normo- or hyperglycaemic conditions in the presence or absence of oxLDL (150 μg/mL) for 3 h. Caspase-3 activity was measured. * *p* < 0.05, ** *p* < 0.01, comparisons between indicated groups. n.s., not significant; IMS32, immortalised mouse Schwann; TLR4, toll-like receptor 4.

We further examined the activity of caspase-3, the primary mediator of apoptosis, which acts downstream of TLR4 signalling, to evaluate the effect of TLR4 inhibition on oxLDL-induced cell death. The findings on caspase-3 activities are consistent with the results shown in Figure 3. The oxLDL treatment did not induce caspase-3 activation, and TAK-242 pre-treatment did not significantly affect caspase-3 activity in the NG group (Figure 5, NG oxLDL(−) TAK-242(−), NG oxLDL(+) TAK-242(−), NG oxLDL(+) TAK-242(+); 1.00 ± 0.06, 0.90 ± 0.04, 0.97 ± 0.02). In contrast, in the HG group, oxLDL treatment significantly increased caspase-3 activity, which was reduced by TAK-242 pre-treatment (Figure 5, HG oxLDL(−) TAK-242(−), HG oxLDL(+) TAK-242(−), HG oxLDL(+) TAK-242(+); 1.04 ± 0.06, 1.30 ± 0.05, 1.01 ± 0.02). Hence, these findings indicate that hyperglycaemia and oxLDL treatment induce the hyperactivation of TLR4 signalling, leading to Schwann cell apoptosis.

## 4. Discussion

We report, for the first time, that exposure to hyperglycaemia induces a synergistic effect on oxLDL-induced apoptosis via the TLR4 pathway in Schwann cells. Elevated glucose and oxLDL levels are more deleterious than either alone. The combination of HG conditions and oxLDL treatment, both associated with the progression of diabetes, may contribute to Schwann cell apoptosis via the hyperactivation of TLR4, leading to neuronal dysfunction in diabetic neuropathy. TLR4 expression is increased in a wide range of cell types such as renal proximal tubule cells, retinal endothelial cells, and monocytes from patients with diabetes [18,19,24]. Additionally, oxLDL contributes to neuronal cell apoptosis via TLR4 or lectin-like oxLDL receptor-1 [13,20]. Hence, oxLDL exerts neurotoxicity, and the expression of its receptor, TLR4, is systemically increased in the diabetic state. However, the relationship between HG conditions and elevated oxLDL levels in the pathogenesis of diabetic neuropathy remains elusive.

In this study, cells were treated with 5 or 25 mM of glucose (equivalent to 90 or 450 mg/dL), which corresponds to normal physiological levels or uncontrolled diabetes, diagnosed as severe diabetic ketoacidosis (>250 mg/dL) and hyperosmolar hyperglycaemic state (>600 mg/dL) [25]. The plasma oxLDL level in subjects with metabolic syndrome has been reported to be 1.45 ± 0.82 mg/dL (equivalent to 14.5 μg/mL), which is lower than the concentration used in our experiments [26]. However, the local physiological concentration in patients with atherosclerosis reached nearly 70 times higher than plasma levels [27]. In our experiments, we used 150 and 300 μg/mL of oxLDL, which also covers physiologically possible concentrations in vivo as described previously [28].

TLR4 was originally identified as a receptor for endotoxins, such as lipopolysaccharide, for defence against microbial infection [29]. In cultured Schwann cells, TLR4 regulates cell proliferation, migration, and apoptosis; however, little is known regarding the physiological and pathophysiological roles of TLR4 in neuronal cells [12]. There is a growing body of evidence suggesting that oxLDL induces neuronal damage in vitro and in vivo, and its concentration is elevated in diabetes [13,16,17,20]. In the present study, oxLDL treatment reduced Schwann cell viability, which was significantly lower in the HG group than in the NG group (Figure 1). We examined the expression of TLR4, which recognises oxLDL and induces cell death in diverse cell types [13,14,15]. The gene and protein expression levels of TLR4 were synergistically elevated by the combination of hyperglycaemia and oxLDL treatment (Figure 3). Hyperglycaemia reportedly increased TLR4 expression in monocytes and renal proximal tubular cells [19,30]. In contrast, oxLDL treatment increased the expression of TLR4 via a positive feedback mechanism [31]. Thus, we postulated that the synergistic induction of TLR4 and its activation by hyperglycaemia and oxLDL treatment could be a potential mechanism of neuronal injury in diabetes. We showed that increased oxLDL-induced cell death under hyperglycaemic conditions was ameliorated by the inhibition of TLR4 signalling (Figure 4). Previous studies found that oxLDL treatment induced caspase-3-dependent apoptosis via the TLR4 pathway and that TLR4 signal inhibition reduced apoptosis in cultured dorsal root ganglia and cardiomyocytes [13,14]. In the present study, oxLDL treatment significantly increased apoptotic caspase-3 activity in the HG group, but not in the NG group, and this increase was abolished by TLR4 signal inhibition (Figure 5). Therefore, these findings suggest that TLR4 hyperactivation leads to neuronal dysfunction in diabetes complicated by dyslipidaemia and elevated circulating oxLDL levels through Schwann cell injury.

Although the pathogenetic role of TLR4 in diabetic neuropathy remains to be established, polymorphisms of TLR4 have been reported to reduce the prevalence of neuropathy in type 2 diabetes [32]. Additionally, previous clinical studies, including the EURODIAB Prospective Complications Study, have shown a close relationship between lipid profiles (such as LDL cholesterol and triglyceride levels) and the risk of diabetic neuropathy [16,33]. Given that oxLDL and saturated fatty acids derived from LDL and triglycerides can act as ligands for TLR4, lipid abnormalities may be involved in the early onset and development of diabetic neuropathy via the TLR4 pathway [13,14,15,34,35]. Interestingly, it has been also reported that atherogenic oxLDL and Alzheimer’s disease peptide β-amyloid trigger proinflammatory responses via the formation of a CD36-TLR4-TLR6 signalling complex in a shared pathway [36]. These observations and our results strongly suggest that the hyperactivation of TLR4 is a possible mechanism underlying the pathogenesis of neuropathy in diabetes complicated by dyslipidaemia. However, further studies are required to elucidate the detailed mechanisms and possible endogenous TLR4 ligands that contribute to the pathogenesis and progression of diabetic neuropathy.

A possible limitation of our study was the LDL oxidation procedure. We used CuSO_4_ for oxLDL preparation, which is the most widely used method for oxLDL preparation in vitro. However, LDL oxidised with copper may not reproduce the complete features of oxLDL generated in vivo as described previously [37]. In addition, our findings are limited to a cellular model; further in vivo experiments are required to fully establish the significance of TLR4 signalling in the aetiology of diabetic neuropathy.

The dysregulation of TLR4 signalling in diabetes complicated by dyslipidaemia may contribute to the pathogenesis and exacerbation of diabetic neuropathy, and TLR4 can be a therapeutic target for diabetic neuropathy.

## Figures and Tables

**Table 1:** Primers used for quantitative real-time PCR.

Genes
ACTB Forward: → 5′-CAT TGC TGA CAG GAT GCA GAA GG-3′
Reverse: → 5′-TGC TGG AAG GTG GAC AGT GAG G-3′
TLR4 Forward: → 5′-TCC CTG CAT AGA GGT AGT TCC-3′
Reverse: → 5′-TCC AGC CAC TGA AGT TCT GA-3′

## Data Availability

Data are contained within the article.
